# Engineering sucrose metabolism in *Pseudomonas putida* highlights the importance of porins

**DOI:** 10.1111/1751-7915.13283

**Published:** 2018-05-28

**Authors:** Hannes Löwe, Peter Sinner, Andreas Kremling, Katharina Pflüger‐Grau

**Affiliations:** ^1^ Systems Biotechnology Technical University of Munich 85748 Garching Germany

## Abstract

Using agricultural wastes as a substrate for biotechnological processes is of great interest in industrial biotechnology. A prerequisite for using these wastes is the ability of the industrially relevant microorganisms to metabolize the sugars present therein. Therefore, many metabolic engineering approaches are directed towards widening the substrate spectrum of the workhorses of industrial biotechnology like *Escherichia coli*, yeast or *Pseudomonas putida*. For instance, neither xylose or arabinose from cellulosic residues, nor sucrose, the main sugar in waste molasses, can be metabolized by most *E. coli* and *P. putida* wild types. We evaluated a new, so far uncharacterized gene cluster for sucrose metabolism from *Pseudomonas protegens* Pf‐5 and showed that it enables *P. putida* to grow on sucrose as the sole carbon and energy source. Even when integrated into the genome of *P. putida*, the resulting strain grew on sucrose at rates similar to the rate of the wild type on glucose – making it the fastest growing, plasmid‐free *P. putida* strain known so far using sucrose as substrate. Next, we elucidated the role of the porin, an orthologue of the sucrose porin ScrY, in the gene cluster and found that in *P. putida*, a porin is needed for sucrose transport across the outer membrane. Consequently, native porins were not sufficient to allow unlimited growth on sucrose. Therefore, we concluded that the outer membrane can be a considerable barrier for substrate transport, depending on strain, genotype and culture conditions, all of which should be taken into account in metabolic engineering approaches. We additionally showed the potential of the engineered *P. putida* strains by growing them on molasses with efficiencies twice as high as obtained with the wild‐type *P. putida. *This can be seen as a further step towards the production of low‐value chemicals and biofuels with *P. putida* from alternative and more affordable substrates in the future.

## Graphical abstract

The article deals with the evaluation of a new, so far uncharacterized gene cluster from *Pseudomonas protegens* Pf‐5 to confer sucrose metabolism to *P. putida*. This metabolic engineering approach resulted in the fastest growing, plasmid‐free *P. putida* strain known so far using sucrose as substrate, unlocking new cheap substrates like molasses. Additionally, we could show that the outer membrane presents a considerable barrier for sucrose transport, as the native, constitutively expressed porins are not sufficient to allow unlimited growth on sucrose. We discuss the importance of considering the outer membrane barrier for metabolic engineering approaches.

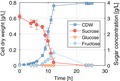

## Introduction

Second‐generation biofuels have received much attention in recent years. Using waste biomass instead of sugar from edible crops, it has been possible to uncouple biofuel production from food production. Therefore, the development of an affordable process using agricultural waste material is generally perceived to be one of the ‘holy grails’ of industrial biotechnology (Sparks and Payne, 2010; Money, 2018). Consequently, it is of great interest to make these carbon sources available to production strains like *Saccharomyces cereivisiae*,* Escherichia coli* or *Pseudomonas putida* in order to maximize the overall yield.

*Pseudomonas putida* is an emerging chassis for industrial biotechnology and a promising host for the production of biofuels and chemicals due to its intrinsic robustness to various sources of stress and its solvent resistance (Ramos *et al*., 2015). Recently, *P. putida* was successfully employed to use aromatic, lignin‐derived compounds (Olivera *et al*., 1998; García *et al*., 1999). Metabolic engineering approaches to expand the substrate spectrum of this organism have already unlocked the hemicellulose monomers D‐xylose and L‐arabinose (Meijnen *et al*., 2008; Dvorak and de Lorenzo, 2018), D‐cellobiose (Dvorak and de Lorenzo, 2018) as well as sucrose (Löwe *et al*., 2017b), the main sugar of molasses from sugarcane and beet, to be used as a carbon source. To confer sucrose metabolism to *P. putida,* genetic constructs were designed based on the *csc* operon from *E. coli* W. *P. putida* EM178 showed reasonable growth on sucrose, when the genes were expressed from a plasmid (Löwe *et al*., 2017b). The *csc* gene cluster in *E. coli* W consists of four genes: *cscA* encoding an invertase (CscA), *cscB* coding for a sucrose/H^+^ symporter (permease CscB), *cscR* encoding a regulator protein (CscR) and *cscK* coding for a fructokinase (CscK). It was shown that *cscA* and *cscB* were sufficient for efficient utilization of sucrose in *E. coli* (Sabri *et al*., 2013). However, when these two genes were integrated into the *P. putida* genome via a mini‐Tn5 transposon, growth on sucrose was slower (μ = 0.27 h^−1^) compared with growth on a glucose/fructose mixture (μ = 0.45 h^−1^). This effect was attributed to poor transport across the membrane of *P. putida* (Löwe *et al*., 2017b). We hypothesized that this might be the result of a lack of sucrose diffusion through the outer membrane or incompatibility of the *E. coli* sucrose permease CscB with *P. putida*. In order to circumvent this bottleneck and to confer efficient sucrose metabolism to *P. putida*, we set out to explore genes from donors closer to *P. putida* than *E. coli*.

For any metabolic engineering strategy aiming to make a new substrate available to Gram‐negative bacteria, one has to consider three barriers that the new substrates have to pass: (i) crossing the outer membrane, (ii) uptake into the cytoplasm and (iii) entry into the metabolism. Most studies focus on the latter two points because transport across the outer membrane is rarely regarded as a problem in model organisms like *E. coli*. However, the outer membrane of *Pseudomonads* is structured differently: Instead of relying on constitutively expressed generalistic porins like *E. coli*'s OmpF and OmpC, *P. putida* and *P. aeruginosa* have a more specialized set of porins and the outer membrane is generally less permeable (Yoshimura and Nikaido, 1982; Nakae *et al*., 1989; Saravolac *et al*., 1991).

In this study, we describe a new approach to tackle all three barriers mentioned above at once. This is achieved by integrating the genes of a so far unannotated operon from *Pseudomonas protegens* Pf‐5 containing a sucrose hydrolase, permease and a sucrose‐specific porin into *P. putida*. We subsequently evaluated the growth behaviour of the resulting strains with sucrose as substrate, thereby paving the way to using sugarcane molasses as a cheap carbon source. In the conclusion, we discuss some general aspects of strategies for metabolic engineering in *Pseudomonads*, taking the outer membrane into account.

## Results and Discussion

### Identification and cloning of an unannotated gene cluster from *P. protegens P*f‐5

**Figure 1:**
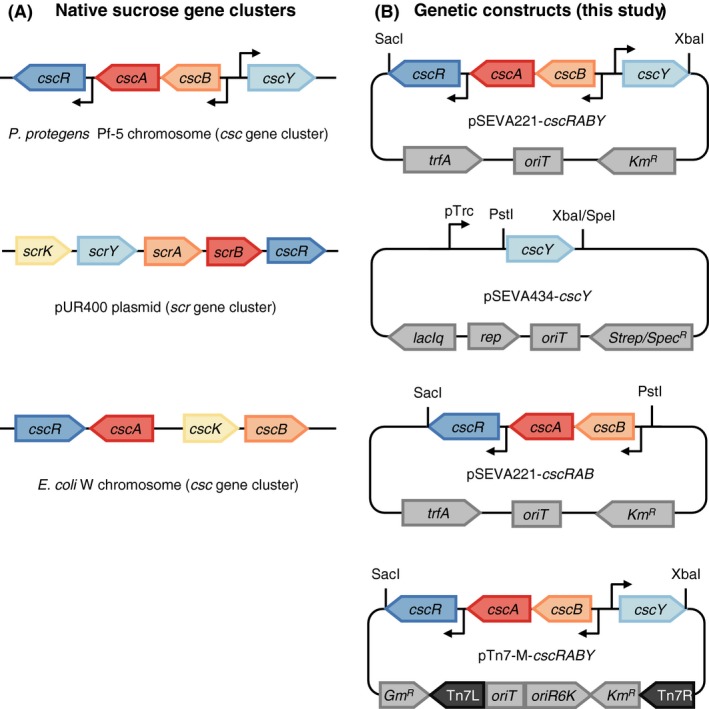
Organization of well‐characterized, native sucrose operons (panel A) and genetic constructs created in this study (panel B). The arrangement of the *csc* gene cluster of *P. protegens* Pf‐5 was taken from pseudomonas.com (Winsor *et al*., 2016), the pUR400 plasmid from (Ebner *et al*., 1988) and the *E. coli* W chromosome from (Sabri *et al*., 2013). A complete list of genetic constructs and their features are listed in Table S1.

The most intensively studied operons for sucrose metabolism in Gram‐negative bacteria are the *scr* genes in the pUR400 plasmid from *Salmonella* (Ebner *et al*., 1988) and the *csc* genes from the enteric bacterium *E. coli* W. The gene clusters are depicted in Fig. 1A. Comparing both sucrose uptake systems, two major differences are observed: First, the ScrAY uptake system transports sucrose via the phosphotransferase system (PTS), thereby phosphorylating the sugar during uptake. In contrast, the transport system of the *csc* genes is driven by the sucrose/H^+^ symporter CscB that facilitates gradient‐driven sucrose uptake. Second, the *scr* gene cluster contains a gene encoding the sucrose‐specific porin ScrY while a homologue is missing in the *csc* genes of *E. coli* (Fig. 1A).

A gene cluster was identified in *P. protegens* Pf‐5 comprising the genes PFL_3236 to PFL_3239 via BLAST homology search (Altschul *et al*., 1990) using *cscB* as query. The genes are annotated as a LacI‐like repressor (PFL_3236), a sucrose hydrolase (PFL_3237), a sucrose or galactoside permease (PFL_3238, with homology to *cscB*), and a sucrose porin (PFL_3239, with homology to *ScrY*). Interestingly, this newly identified gene cluster of *P. protegens* Pf‐5 carries features of both known gene clusters because a gene encoding a non‐PTS permease resembling the CscB protein of *E. coli* W, as well as a gene coding for a porin resembling ScrY, is present. Due to the high similarity between PFL_3236 and *cscB*, we hypothesized that the corresponding proteins carry out the same function and that the whole operon is responsible for sucrose uptake and hydrolysis. Apart from *P. protegens* Pf‐5, there are a few other *Pseudomonas* strains that carry orthologues of the *cscB* gene encoding the proton‐gradient‐driven sucrose transporter in their genomes. For instance, *Pseudomonas fluorescence* strains AU13852 and AU20219 posses a gene cluster with the same genetic organization as found in *P. protegens* Pf‐5 (Fig. S3). As an analogy to the *csc* operon of *E. coli* W, we will denominate the genes of the *P. protegens* Pf‐5 cluster in the rest of the article as *cscR* (repressor, PFL_3236), *cscA* (sucrose hydrolase, PFL_3237), *cscB* (permease, PFL_3238) and *cscY* (porin, PFL_3239) making the whole cluster *cscRABY* (Fig. 1A).

To test the above mentioned hypothesis that the *cscRABY* operon is responsible for sucrose uptake and hydrolysis and to elucidate in more detail the function of the porin CscY of the *P. protegens* Pf‐5 gene cluster, the whole fragment (~6 kB) was cloned into pSEVA221 without the addition of any promoter sequence, as the native promoters are predicted to be in the middle of the fragment (Fig. 1B). This should leave the regulation as in its native host, thereby avoiding the need to express the operon in the presence of sucrose and preventing the associated metabolic stress when other substrates are used. In scenarios where only sucrose is to be used, removal of the regulation or use of tailored promoters should be considered.

Additionally, the same fragment without the last gene encoding the porin CscY was likewise inserted into pSEVA221 (Fig. 1B; see Experimental Procedures for details). For complementation experiments, an expression vector (based on pSEVA434) containing only the gene c*scY* was created. We also constructed the pTn7‐M‐based plasmid carrying the whole operon to test the functionality of the *cscRABY* operon of *P. protegens* Pf‐5 when present in single copy and to allow stable integration into *P. putida* without the need for selective pressure. Conjugation and integration of this mini‐Tn7‐based transposon into *P. putida* EM178 yielded *P. putida att*Tn7::*cscRABY*.

### The *cscRABY* genes conferred the ability to metabolize sucrose, but not maltose or lactose

**Figure 2:**
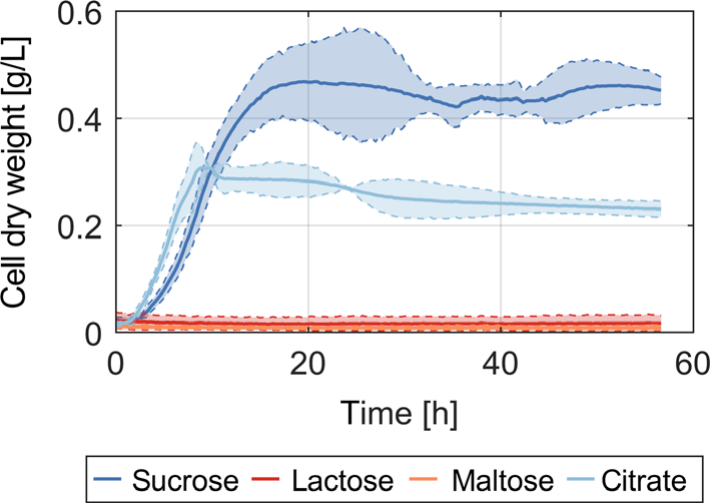
Cell dry weights of *P. putida* EM178 (pSEVA221‐*cscRABY*) on sucrose over time. *P. putida* EM178 (pSEVA221‐*cscRABY*) was grown in the presence of 3 g l^−1^ of sucrose (dark blue), lactose (red), maltose (orange) or citrate (light blue) in a microplate reader in M9 minimal medium. The shaded areas represent the 95% confidence bands, which were estimated from three replicates.

First, we tested whether the new gene cluster was able to give *P. putida* the ability to grow on sucrose as the sole carbon source. As the putative sucrose permease CscB (PFL_3238) also shows homology to a galactoside permease (Accession number EFK49434.1, *E*‐score 4e‐71) from *E. coli* and belongs to the LacY super‐family, we also tested whether the common disaccharides lactose and maltose could serve as a carbon source in the presence of pSEVA221‐*cscRABY*. All three substrates cannot be metabolized by native *P. putida* EM178, albeit they might be transported by CscB. The results of cultivations in M9 medium using 3 g l^−1^ of either of the three disaccharides or citrate as positive control are illustrated in Fig. 2. The only disaccharide able to support the growth of *P. putida* was sucrose. Moreover, the high growth rate of 0.292 ± 0.016 h^−1^ indicates sucrose as the preferred substrate. In contrast, the strain bearing only the three genes *cscRAB* was not able to grow after 3 days of cultivation in M9 with sucrose (data not shown). Therefore, it was tempting to speculate that the porin CscY carried out a vital role during the consumption of sucrose by *P. putida*, which will be addressed in the following section. Citrate was used at the same (mass) concentration as sucrose, however, it showed lower yields in this experiment, which can be attributed to its higher degree of oxidation.

The permease CscB also has high similarity to a raffinose permease from *E. coli* (Accession number EGI19556.1, *E*‐score 9e‐81). As raffinose and sucrose are structurally related and share the same sucrose (sub)unit, we cannot exclude that the gene cluster might also be able to transport and cleave raffinose. However, a second hydrolase may be needed for efficient cleavage of raffinose for the d‐galactopyranosyl‐(1→6)‐α‐d‐glucopyranoside moiety.

### Expression of CscY complemented the loss of growth in *P. putida* pSEVA221‐*cscRAB*

**Figure 3:**
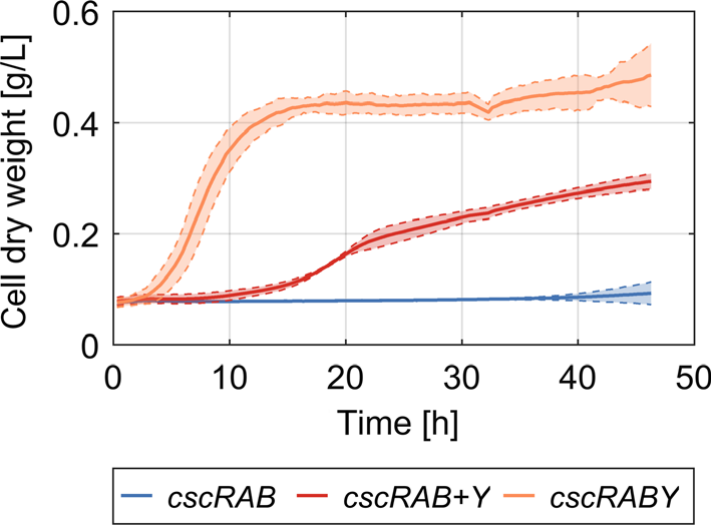
Complementation studies with *P. putida* EM178. The strain without the porin (pSEVA221‐*cscRAB*) was complemented with a LacI/P_trc_ promoter‐controlled expression vector containing the gene encoding the porin CscY (pSEVA434‐*cscY*). Growth on sucrose of the different strains was monitored in a microplate reader. The plasmid pSEVA221‐*cscRABY* was used as a positive control and the empty expression vector pSEVA434 as a negative control. The mean values of nine replicates (three biological with three technical replicates each) and 95% confidence bands were estimated from all replicates.

The next step was to analyse the role of the porin CscY in more detail, as we had indications from earlier studies that the transport of sucrose across the membrane might be the limiting step (Löwe *et al*., 2017b). To test this assumption, we compared the growth of *P. putida* (pSEVA221‐*cscRABY)* and *P. putida* (pSEVA221‐*cscRAB*) on sucrose (Fig. 3). In fact, no growth could be observed on sucrose in the absence of CscY. To confirm that this effect was caused by the absence of the porin and was not a consequence of changes in the promoter region of *cscRAB* as a cloning artefact, *P. putida* (pSEVA221‐*cscRAB*) was complemented with the expression plasmid pSEVA434‐*cscY*. As can be seen in Fig. 3, the plasmid pSEVA434‐*cscY* was indeed able to restore the loss of growth associated with a lack of the porin in pSEVA221‐*cscRAB*, albeit at a slower rate than with the full set of genes. The experiment in Fig. 3 was performed in a microplate reader with LB as the preculture medium. The same experiment with identical precultures was also carried out in shaking flasks yielding similar results (Fig. S1 and Table 1). Following the sugar concentration in the medium over time revealed that sucrose was taken up and split only by strains expressing the porin (Fig. S1).

**Table 1:** Relevant growth parameters of strains constructed in this study

Genotype *P. putida* EM178	Substrate	Growth rate μ [h^−1^]	Biomass yield^a^ *Y*_*X/S*_ [g/g]	Substrate uptake rate^b^ *q* [mmol g_CDW_^−1^ h^−1^]
Wild type	Sucrose	n.d.	n.d.	n.d.
*att*Tn7::*cscRABY*	Sucrose	0.45 ± 0.02	0.23 ± 0.02	5.9 ± 0.7
Wild type	Sugarcane molasses	0.202 ± 0.018	0.052^c^	n.m.
*att*Tn7::*cscRABY*	Sugarcane molasses	0.265 ± 0.017	0.125^c^	n.m.
pSEVA221‐*cscRABY*	Sucrose (LB glucose preculture)	0.3945 ± 0.0001	0.277 ± 0.013	4.16 ± 1.9
pSEVA221‐*cscRAB*	Sucrose (LB glucose preculture)	n.d.	n.d.	n.d.
pSEVA221‐*cscRAB +* pSEVA434‐*cscY*	Sucrose (LB glucose preculture)	0.059 ± 0.013	0.17 ± 0.04	1.0 ± 0.3
pSEVA221‐*cscRABY*	Sucrose (M9 glucose preculture)	0.515 ± 0.005	n.m.	n.m.
pSEVA221‐*cscRAB*	Sucrose (M9 glucose preculture)	0.470 ± 0.019	n.m.	n.m.
pSEVA221‐*cscRAB +* pSEVA434‐*cscY*	Sucrose (M9 glucose preculture)	0.38 ± 0.02	n.m.	n.m.

n.d., not detectable; n.m., not measured.

**a**. Yields were calculated from three averaged replicates of cell dry weight and substrate concentrations; errors are calculated from regression.

**b**. Sucrose uptake rates were calculated from growth rates and biomass yields; errors were calculated with Gaussian propagation of uncertainty. The values in this table consider the total amount of sucrose metabolized – glucose and fructose in the supernatant were considered to be not taken up.

**c**. Biomass yields in experiments using sugarcane molasses as a substrate were calculated from maximal cell dry weights and are related to the concentration of molasses (10 g l^−1^) and not to the sugar content as molasses are constituted by three different sugars.

The unbalanced expression of the porin might provide an explanation for the slower growth rate of the strain complemented with CscY (see Fig. 3): The backbone plasmid pSEVA434 with its pBBR1 ori (moderate copy number) and the control of expression by the leaky LaqI/P_trc_ promoter (Balzer *et al*., 2013) might produce CscY at an unfavourably high rate. Furthermore, the artificial expression of membrane proteins is known to have a negative influence on cell vitality (Wagner *et al*., 2007). In fact, formation of flocks – probably biofilm formation, a common stress response in *P. putida* – was observed in the strains expressing CscY from pSEVA434*‐cscY* in shaking flasks. This was accompanied by a reduction in the cell dry weight of the planktonic cells (compare Fig. S1).

From these experiments, we concluded that, at least under these experimental conditions, the native outer membrane of *P. putida* was not permeable to sucrose. Compared to *E. coli* where transport of small molecules across the outer membrane is mainly facilitated by rather unspecific porins, *P. putida* has an outer membrane that more closely resembles the membrane of *P. aeruginosa*: homologues of the unspecific porins from *E. coli* (OpmF and OmpC) are not present. Instead, the general porin is OprF, which has a diffusion rate of two orders of magnitude lower than OpmF and OmpC of *E. coli* (Saravolac *et al*., 1991; Nikaido, 2003; Eren *et al*., 2012). This lower permeability is believed to be a major contributor to the remarkable resistance to toxic agents in fluorescent *Pseudomonads* (Nikaido, 2003) and could also be important for *P. putida*'s resistance to chemical stresses.

### Sucrose transport across the outer membrane was dependent on preculture medium

While carrying out the complementation experiments, we realized that, when using M9 medium‐grown precultures instead of LB‐grown precultures for otherwise identical experiments, the growth of *P. putida* (pSEVA221‐*cscRAB*) was not impaired on M9 sucrose, but the cells grew at almost the same rate as *P. putida* (pSEVA221‐*cscRABY*) (Fig S2 and Table 1). This unexpected finding might be explained by the different metabolic regimes of the cultures: Cells grown in minimal medium with only glucose as a carbon source have a glycolytic regime whereas those grown in LB need to carry out gluconeogenesis. For these different lifestyles, a different set of proteins and also a different composition of the outer membrane is required, which depends on the culture environment (Thompson *et al*., 2010; Choi *et al*., 2014). We speculate that the outer membrane of cells grown in M9 medium has a different set of outer membrane proteins and might already possess sucrose transport activity, whereas the one of LB‐grown cells does not. To test this hypothesis, we conducted a periplasm swelling experiment that is based on a method described by Nakae *et al*. (1989). Cells were grown in different media (LB, M9 with glucose, or M9 with citrate) and subsequently washed with a hypertonic solution containing glycerol (which should pass the outer membrane), sucrose and xylose. The substances that passed the outer membrane should be present in the periplasm after this first washing step.

**Figure 4:**
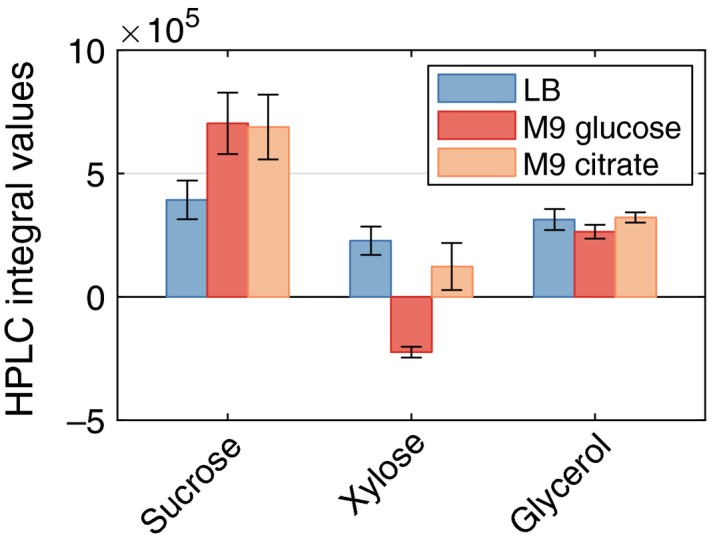
Permeability of the outer membrane of *P. putida* EM178 estimated from the swelling experiment using a hypertonic solution of 0.15 M sucrose, xylose and glycerol in M9 medium. The bars represent the estimated substrate amounts (as quantified by their peak areas in HPLC) after washing the cells with deionized water, normalized with the fluorescence signal of GFP in supernatants according to Eqs (1) and (2). They should therefore represent the amount of substrate that is able to permeate through the outer membrane. Mean values and standard deviations are derived from three replicates. From left to right: blue bars: precultures grown in LB; red bars: precultures grown in M9 glucose; orange bars: precultures grown in M9 citrate.

Subsequently, these compounds now present in the periplasm were washed out again in a second washing step with deionized water. Then, the sugar concentrations in the supernatant were determined, which allowed us to draw conclusions on the permeability of the outer membrane for the detected sugars. As expected, the glycerol concentration was independent of preculture conditions, corroborating the assumption that it can diffuse freely across the membrane. However, sucrose was twice as concentrated in the supernatants of cells grown in M9 medium (Fig. 4). This can be explained by the faster diffusion rate of sucrose through the outer membrane of M9‐grown cells compared with LB‐grown cultures and was a further sign that using LB as preculture medium led to a lower permeability of the outer membrane for sucrose. The negative values for Xylose in Fig. 4 are an artefact from the calculation (Eq. (2)) and indicate that xylose is metabolized by the cells. In fact, the loss of xylose in the chromatogram is accompanied by the appearance of another peak close by, which is very likely an oxidized intermediate (data not shown). This behaviour of xylose was also reported by (Dvorak and de Lorenzo, 2018) and makes it an unsuitable internal standard for swelling experiments like this. Therefore, GFP and glycerol were used as internal standards.

The reason for the differences in the uptake of substrates through the outer membrane can probably be attributed to a different set of porins as a result of the preculture medium. In fact, previous proteome studies showed a strong dependency of most outer membrane porins on medium composition and carbon source in *P. putida* F1 and KT2440 (Thompson *et al*., 2010; Choi *et al*., 2014). From our experiments, we cannot conclude which particular porin might be responsible for sucrose uptake, but outer membrane fractions and purified porins of other *Pseudomonads* have been shown to be able to transport sucrose to a certain extent (Trias *et al*., 1988; Shrivastava *et al*., 2011; van den Berg, 2012). The major outer membrane porin of *P. aeruginosa* is OprF. As *P. putida* and *P. aeruginosa* share similar features in the organization of their outer membrane (Saravolac *et al*., 1991) and OprF is able to transport molecules as large as raffinose (Nikaido, 2003), it might well be responsible for sucrose transport in this work.

In any case, the whole *cscRABY* operon was used to construct a sucrose‐consuming *P. putida* strain to avoid phenotypic variations and to ensure sucrose uptake independent of the preculture medium.

### Genomic integration of *cscRABY* genes resulted in stable and rapid sucrose‐dependent growth

**Figure 5:**
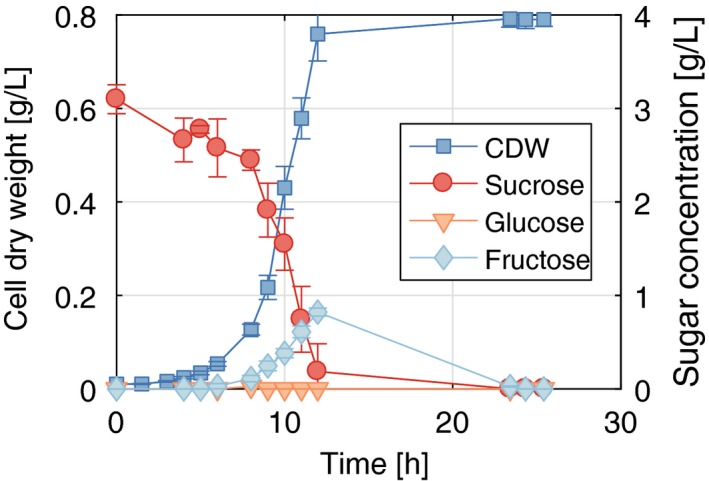
Cell dry weight and sugar concentrations of a culture of *P. putida* EM178 *att*Tn7::*cscRABY* over time*. *Cells were grown in M9 medium supplemented with 3 g l^−1^ sucrose. Mean values and standard deviations of three independent biological replicates are shown. Experiments were performed in shaking flasks filled with 50 mL liquid and at an agitation rate of 220 rpm at 30°C.

As a next step, we aimed to integrate the gene cluster into the chromosome of *P. putida* in order to avoid the need for a plasmid and the associated antibiotic selection pressure. Using antibiotics may not be desirable or suitable for some applications. Therefore, we integrated the *cscRABY* genes into the *att*Tn7‐site of *P. putida* EM178 via the mini‐Tn7 transposon vector pTn7‐M and evaluated the growth of the resulting strain on sucrose (Fig. 5). In shaking flasks growth rates of 0.45 ± 0.02 h^−1^ and sucrose uptake rates of 5.9 ± 0.7 mmol g_CDW_^−1^_ _h^−1^ (calculated from three replicates) could be reached. For a more detailed overview of the growth rates, yields and substrate uptake rates, the key growth parameters are listed in Table 1. This compares well to growth rates measured with the monomers glucose and fructose and is around 60% higher than previously reported growth rates (Löwe *et al*., 2017b). Time courses of cell dry weight and sugar concentration in the supernatant are depicted in Fig. 5. Interestingly, fructose accumulated in the growth medium during fermentation, which might be explained by leakage of fructose after intracellular splitting of sucrose or by extracellular hydrolase activity. The latter option is not very likely because no splitting activity could be measured when the porin was lacking (Fig. S1). Leakage of fructose is also supported by the fact that *P. putida* does not show any fructokinase activity (Sawyer *et al*., 1977). Instead, fructose is phosphorylated during uptake through the native phosphotransferase system (PTS^Fru^) (Velázquez *et al*., 2007). In fact, the absence of FruB, the cytoplasmatic component of the PTS^Fru^, completely abolishes growth on fructose (Velázquez *et al*., 2007). Thus, after the intracellular cleavage of sucrose into glucose and fructose, glucose could be metabolized directly by glucokinase Glk, whereas fructose has to be secreted and taken up again via the fructose‐PTS system in order to be accessible to *P. putida*'s metabolism. Alternatively, glucose could also be secreted and then oxidized in to gluconate before uptake. We did not measure any relevant amount of intermediates of glucose metabolism via HPLC, which indicates that glucose either did not accumulate because of rapid oxidation and re‐uptake or never left the cell.

For future applications, it might be worth considering the addition of a functional fructokinase to the metabolism of *P. putida*. This would result in a hybrid system consisting of the *csc* genes from *P. protegens* Pf‐5 and the ones of *E. coli* W that also comprise a fructokinase, and could be a way to make sucrose metabolism more efficient. Engineering the central carbon metabolism of *P. putida* was recently demonstrated (Sánchez‐Pascuala *et al*., 2017) and offers the potential to replace biomodules that are inefficient in an industrial context with streamlined versions for the desired application.

### Cultivation of *P. putida* EM178 *att*Tn7::*cscRABY* in sugarcane molasses

**Figure 6:**
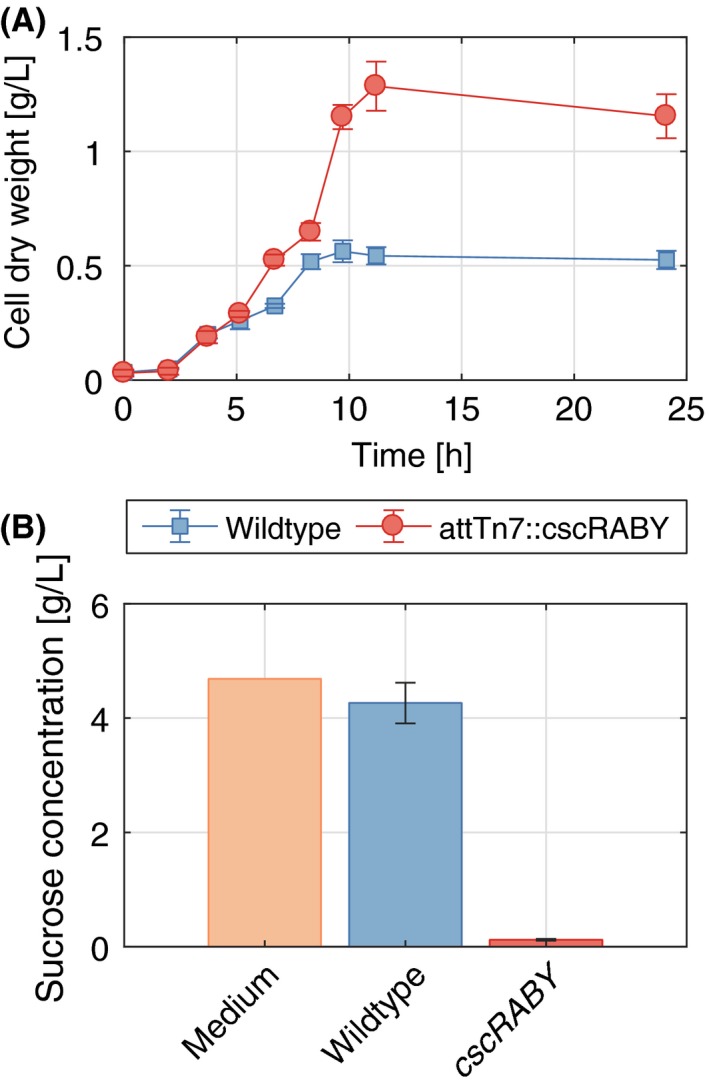
Growth of *P. putida* EM178 *att*Tn7::*cscRABY* and *P. putida* EM178 on molasses. A M9 molasses (10 g l^−1^) medium was used, and experiments were performed in shaking flasks with 50 mL volume in three biological replicates; agitation rate 220 rpm; temperature 30°C. A. Cell dry weight over time and (B) sucrose consumption of both strains, compared with the original concentration in the medium after 24 h.

We cultivated *P. putida* EM178 *att*Tn7::*cscRABY* in M9 medium with untreated sugarcane molasses as a carbon source (Fig. 6) to prove the functionality of the *cscRABY* gene cluster on the industrially relevant waste product sugarcane molasses. *P. putida* EM178 was used as negative control. For the first 5 h, both strains grew nearly identical, but after 9 h the wild‐type strain stopped growing and the final cell dry weight of the engineered strain was more than twice as high as the cell dry weight of the wild type. Sugarcane molasses mainly consist of the carbohydrates sucrose, glucose and fructose. The latter two sugars can also be used by wild‐type *P. putida*, explaining the initial growth of the negative control. Sucrose can only be metabolized by the strain carrying the *cscRABY* genes, which is reflected by higher final cell dry weights (Fig. 6A), as well as almost complete depletion of sucrose in the supernatant (Fig. 6B) and thus higher biomass yields (Table 1). Sugarcane molasses are a very cost effective carbon source, which might help new bioprocesses using *P. putida* to come to life.

## Conclusion

Although a sucrose porin does not seem to be necessary in *E. coli* to efficiently metabolize sucrose (Sabri *et al*., 2013), we found that sucrose is not reliably transported across the outer membrane in *P. putida* unless a suitable porin is expressed. An explanation for that can be found in the lower permeability of the outer membrane of *Pseudomonads* compared with *E. coli*. This remarkable difference has to be taken into account when engineering new pathways in *P. putida* and any other Gram‐negative bacteria where a substrate needs to enter the cell or a product is to be secreted out of the cell. Porins normally do not receive much attention in metabolic engineering, but, depending on the desired metabolites, they might be of great importance. By introducing a suitable porin, productivity can be increased if the uptake of the substrate is the limiting factor. In other cases, a porin might improve cell vitality if it facilitates the passage of a possible toxic product through the outer membrane. In the work described here, we were able to show that additionally to the genes *cscRAB,* the porin CscY was needed in *P. putida* for efficient growth on sucrose when precultures were grown in LB medium.

This highlights the phenotypic instability when the outer membrane is neglected in the engineering process. When the *csc* genes were fully implemented, *P. putida* showed excellent growth with sucrose as the sole carbon and energy sources, even when genomically integrated and thus present in single copy. In addition, biomass yield increased more than twofold when grown on sugarcane molasses compared with the wild type, opening up the possibility to grow *P. putida* at low substrate costs. This is especially interesting for products that have to compete with nonrenewable alternatives like bioplastics or biofuels, but also for products that are already produced with *P. putida* like hydroxystyrene (Verhoef *et al*., 2009), phenylalanine (Molina‐Santiago *et al*., 2016) or catechols (Robinson *et al*., 1992) (for an overview, see (Poblete‐Castro *et al*., 2012; Loeschcke and Thies, 2015)). Medium‐chain‐length polyhydroxyalkanoates, a promising type of bioplastics, can be produced by *P. putida* from fatty acids (Huisman *et al*., 1992) that are a major part of waste streams from oil mills. However, they can also be produced by *P. putida* using glucose (Huijberts *et al*., 1992; Acuña *et al*., 2014) or sucrose (Löwe *et al*., 2017a) as carbon source and metabolic engineering is already applied to improve these processes (Acuña *et al*., 2014). This and other efforts to streamline *P. putida* as a production organism will open up the way for new processes and products that can contribute to making a bio‐based and renewable economy finally come to life.

## Experimental procedures

### Organisms, strains and cultivation

*Escherichia coli* DH5α λ‐*pir* was used for the extraction of plasmids, transformation and as the plasmid donor in conjugation. *E. coli* HB101 (pRK600) and *E. coli* DH5α (pTnS1) served as helpers in conjugation and Tn7 transposition respectively. *P. putida* EM178, a prophage‐free derivative of *P. putida* KT2440 (created at Victor de Lorenzo's lab at CNB, Madrid), was used as a working strain. The complete list of organisms in this study and their origin are given in Table S2.

Cultivation was performed as described previously (Löwe *et al*., 2017b). In brief, LB medium was used in all precultures for genetic manipulations and precultures for shaking flasks experiments if not noted otherwise. M9 minimal medium (Miller, 1972) was used for cultivation experiments with specific carbon sources. Shaking flasks were incubated in an orbital shaker at 220 rpm agitation at 30°C for *P. putida* and 37°C for *E. coli*. Antibiotics were used for selection when necessary (standard concentrations: 50 mg l^−1^ kanamycin, 200 mg l^−1^ streptomycin, 10 mg l^−1^ gentamicin).

A microplate reader (Tecan, Austria) was used for the cultivation at the 200 μl scale. Every 20 min, the microtitre plate with the cultures was shaken for 1 min and the optical density at 600 nm was measured. The temperature was controlled to 30 ± 1°C. Cell dry weights were calculated from optical densities with correlation factors that were determined beforehand in growth experiments of *P. putida* EM178 with M9 glucose medium in shaking flasks.

### Genetic manipulations

All genetic constructs were created by restriction/ligation cloning and are listed in Table S1. The *cscRABY* gene cluster was amplified from genomic DNA of *P. protegens* Pf‐5 using the primers fwP_I_scr_P_pro and rvP_scr_P_pro (compare Table S3). The PCR product was cut with restriction enzymes SacI and XbaI (New England Biolabs, Ipswich, MA, USA) for the full gene cluster and with SacI and PstI to obtain only the region spanning the genes *cscRAB*. These fragments were subsequently ligated into the multiple cloning site of the likewise digested pSEVA221. Plasmid pTn7‐M‐*cscRABY* was created by the digestion of pSEVA221‐*cscRABY* with restriction enzymes SacI and XbaI and insertion into the identically cut multiple cloning site of pTn7‐M. To create pSEVA434‐*cscY*, pSEVA221‐*cscRABY* was digested with PstI and XbaI and ligated into the multiple cloning site of pSEVA434 that was previously cut with PstI and SpeI.

Constructs cloned in *E. coli* were transferred to *P. putida* EM178 via conjugation as described by others (de Lorenzo and Timmis, 1994). The method was simplified as follows: first, 200–300 μl of overnight grown cultures (LB medium) of plasmid donor, helper strain [*E. coli* HB101 (pRK600)] and recipient (*P. putida* EM178) was mixed and subsequently centrifuged (10 min at 8000 *g*), the supernatant was discarded, and the pellet was resuspended in the remaining droplet. It was transferred to LB agar plates without antibiotics and incubated as a sitting drop for 6–10 h. The cells were then simply streaked onto an M9 citrate agar plate with suitable antibiotics.

### Sugar and alcohol determination by HPLC

High‐performance liquid chromatography was used to quantify the substrates and intermediates of sucrose metabolism: sucrose, glucose and fructose. Standards of 2 g l^−1^ were used of each substrate. Glycerol and xylose were only determined semiquantitatively by considering their peak areas. Samples were prepared as follows: Cells were separated from samples by centrifuging at least 400 μl of cultivation broth at 17 000 *g* for 5 min. Supernatants were filtered through 0.22‐μm regenerated cellulose filter plates and injected (20 μl) into the HPLC (Agilent 1100 series, Waldbronn, Germany). Analytes were separated in a Shodex SH 1011 column at a flow rate of 0.45 ml min^−1^ with 0.5 mM sulfuric acid as the mobile phase at 30°C. Concentrations were calculated by integration of the peak area of each peak and correlation to the corresponding standards.

### Outer membrane swelling experiments

Sucrose import into the periplasm of *P. putida* was evaluated with a method relying on membrane swelling experiments, similar to Nakae *et al*. (1989): *P. putida* grown with either LB, M9 glucose or M9 citrate medium was upconcentrated to an optical density of about 200 at 600 nm in a volume of 500 μl. Cells were first washed with 500 μl of phosphate‐buffered saline and in a second step with M9 medium containing 0.15 M of sucrose, xylose and glycerol. Xylose and glycerol were added as internal standards as these two substances could be well separated from sucrose by the HPLC method described above. Additionally, purified eGFP, which should be unable to enter the cell and is easily measurable via its fluorescence, was added at a concentration of 0.0332 g l^−1^ as an internal standard. After washing the culture with this complex mixture, cells should have taken up those substances that could diffuse through the outer membrane because the solution was hypertonic. Next, the cells were washed with 500 μl deionized water which should extract the content of the periplasm into the washing solution. Both, the supernatants after washing with the hypertonic solution and deionized water were taken as samples to measure sucrose, xylose, glycerol and eGFP.

With such a big number of cells, there was an unneglectable amount of residual water (*V*_residual_) after centrifugation which might still contain the components of the hypertonic solution. This was corrected with the residual eGFP signal (F1: signal after hypertonic shock, F2: signal after hypotonic shock):

(1) $$V_{\text{residual}}=\frac{F_{2}}{F_{1}-F_{2}}500\mu l$$

This residual volume contained the concentrations of sucrose, xylose and glycerol as determined by HPLC. After washing the cells with deionized water and centrifugation, the supernatants included the sugars that were washed out of the periplasm and those that were left in the residual water after washing with the hypertonic solution. The concentrations had to be corrected with the volume of the residual water (Eq. (1)) and the concentrations of the substances before (Index 1) and after washing (Index 2) in order to calculate the concentration in the periplasm only. The contents of the periplasm after a hypertonic shock could then be calculated:

(2) $$c_{i,\;\mathrm{periplasm}}=c_{i,\;2}-\frac{V_{\mathrm{residual}}}{V_{\mathrm{residual}}+500\mu l}c_{i,\;1}$$

These were the concentrations that we assumed to be in the periplasm after the hypertonic shock and should give information on the extent of diffusion across the outer membrane. All experiments were performed in three biological replicates.

## Conflict of interest

None declared.

## Supporting information


**Figure S1.** Cell dry weights and sugar concentration over time of different *P. putida* strains in M9 sucrose (3 g l^−1^) in shaking flasks derived from LB pre‐cultures.
**Figure S2.** Cell dry weights over time of different *P. putida* strains in M9 sucrose (3 g l^−1^) in shaking flasks derived from M9 pre‐cultures.
**Figure S3.** Organization of closely related gene clusters in other *Pseudomonads* that also contain a putative sucrose/H+‐symporter CscB.
**Table S1.** Plasmids used and constructed in this work.
**Table S2.** Bacterial strains that were used in this work.
**Table S3.** Oligonucleotides used for PCR reactions in this study with name, sequence and function.
