# Supplementary material for: Engineering sucrose metabolism in Pseudomonas putida highlights the importance of porins
Source: Microb Biotechnol. 2018 May 28;13(1):97–106. doi: 10.1111/1751-7915.13283 (PMC6922520; doi:10.1111/1751-7915.13283)
Supplement: Supplementary file 1 — Figure S1. Cell dry weights and sugar concentration over time of different P. putida strains in M9 sucrose (3 g l−1) in shaking flasks derived from LB pre‐cultures. Figure S2. Cell dry weights over time of different P. putida strains in M9 sucrose (3 g l−1) in shaking flasks derived from M9 pre‐cultures. Figure S3. Organization of closely related gene clusters in other Pseudomonads that also contain a putative sucrose/H+‐symporter CscB. Table S1. Plasmids used and constructed in this work. Table S2. Bacterial strains that were used in this work. Table S3. Oligonucleotides used for PCR reactions in this study with name, sequence and function. [file MBT2-13-97-s001.pdf]

Supporting Information

for

Engineering sucrose metabolism in *Pseudomonas putida* highlights the importance of porins

by

*Hannes Löwe, Peter Sinner, Andreas Kremling, and Katharina Pflüger-Grau**

Associate Professorship of Systems Biotechnology, Technical University of Munich, 85748 Garching (Germany)

Short Title: Porins in sucrose metabolism of *P. putida*

* For correspondence: Katharina Pflüger-Grau, Associate Professorship of Systems biotechnology, Technical University of Munich, Boltzmannstr. 15, 85748 Garching (Germany), Tel.: +49 89 289 15765; Fax.: +49 89 289 15766, email: k.pflueger-grau@tum.de

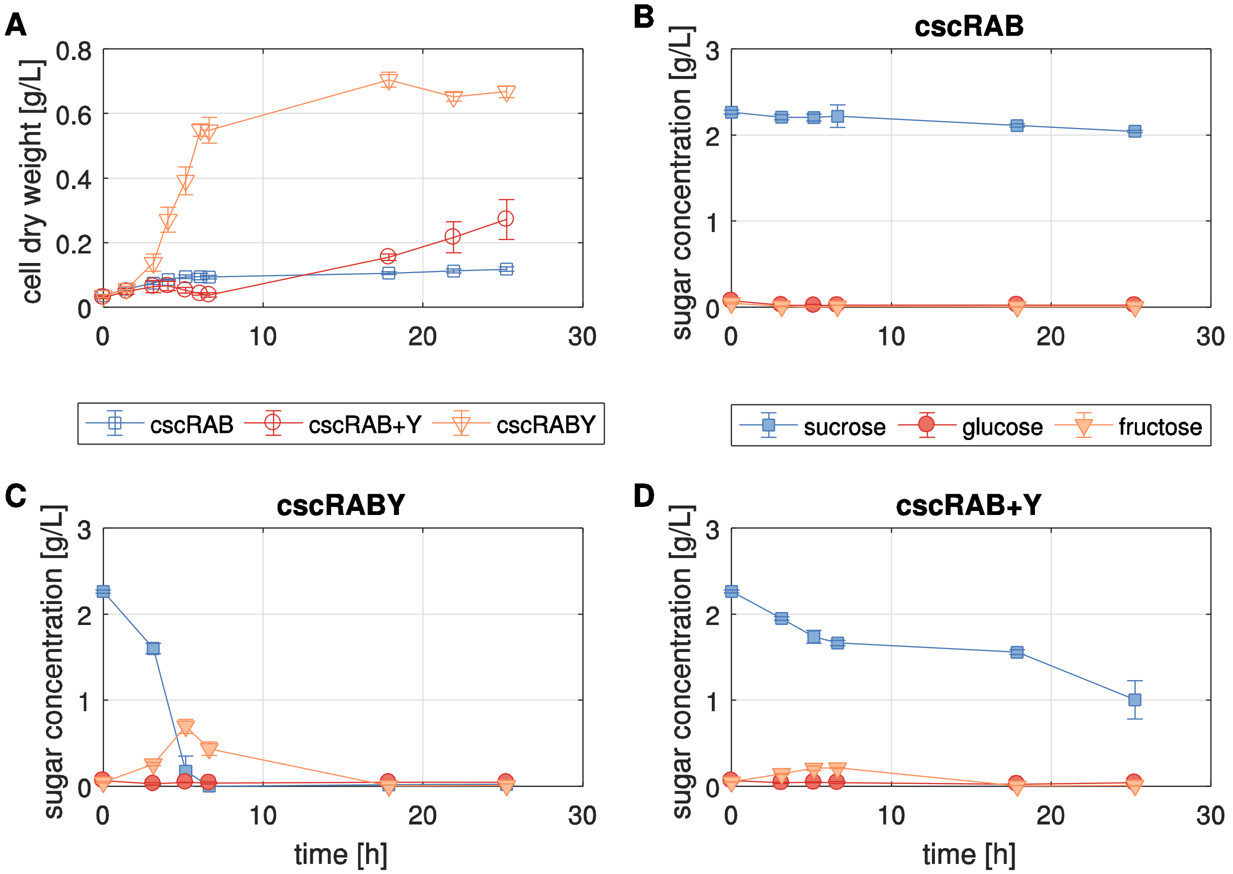

Figure S1: Cell dry weights and sugar concentration over time of different *P. putida* strains in M9 sucrose (3 g/L) in shaking flasks derived from LB pre-cultures. Standard deviations are calculated from three biological replicates. **A)** Cell dry weights of *P. putida* EM178 (pSEVA221-*cscRAB* + pSEVA434), *P. putida* EM178 (pSEVA221-*cscRAB* + pSEVA434-*cscY*) and *P. putida* EM178 (pSEVA221-*cscRABY*); **B)** sucrose concentrations of *P. putida* EM178 (pSEVA221-*cscRAB* + pSEVA434); **C)** sucrose concentrations of *P. putida* EM178 (pSEVA221-*cscRAB* + pSEVA434-*cscY*); **D)** sucrose concentrations of *P. putida* EM178 (pSEVA221-*cscRABY*)

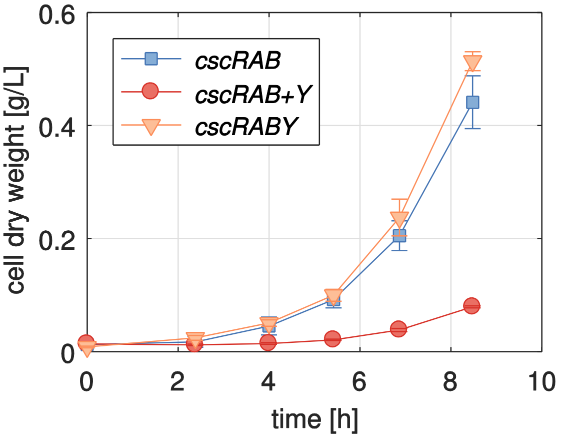

Figure S2: Cell dry weights over time of different *P. putida* strains in M9 sucrose (3 g/L) in shaking flasks derived from M9 pre-cultures. Standard deviations are calculated from three biological replicates. *cscRAB*: *P. putida* EM178 (pSEVA221-*cscRAB* + pSEVA434); *cscRAB+Y*: *P. putida* EM178 (pSEVA221-*cscRAB* + pSEVA434-*cscY*); *cscRABY*: *P. putida* EM178 (pSEVA221-*cscRABY*)

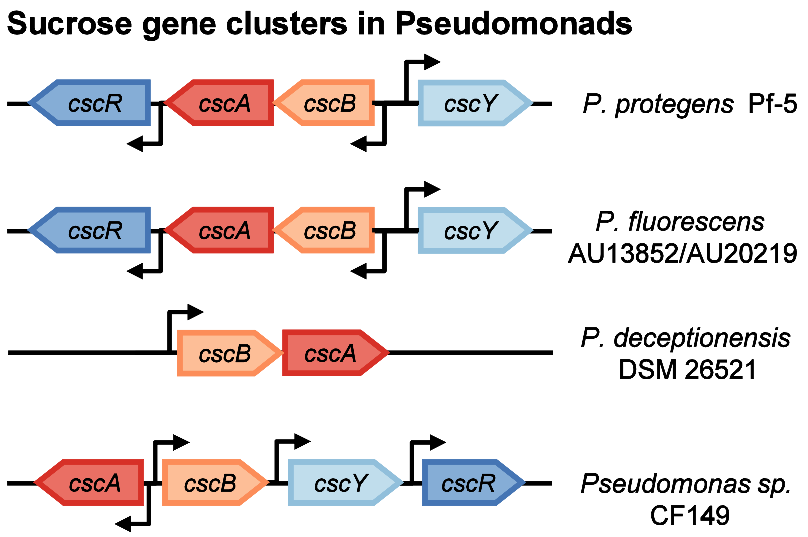

Figure S3: Organization of closely related gene clusters in other *Pseudomonads* that also contain a putative sucrose/H^+-symporter CscB. Promoter position is estimated based on gaps between the genes. Alignments were taken from "pseudomonas.com" (Winsor et al., 2016)

Table S1 | Plasmids used and constructed in this work

Plasmid name	Description	Source or reference
pSEVA434	Cloning vector; pBBR1-ori; Sm/Sp-resistance; IPTG-inducible P_{trc} -promoter	Silva-Rocha *et al.*, 2013
pSEVA221	Cloning vector; R2K-ori; Km-resistance; Empty multiple cloning site	Silva-Rocha *et al.*, 2013
pTn7-M	miniTn7 transposon vector for genomic integration into *att*Tn7-site; R6K-ori; Gm-resistance	Zobel *et al.*, 2015
pSEVA221-*cscRABY*	Derivative of pSEVA221; genes PFL_3236-PFL_3239 from *P. protegens* Pf-5 integrated into MCS with restriction enzymes SacI and XbaI	This work
pSEVA221-*cscRAB*	Derivative of pSEVA221; genes PFL_3236-PFL_3238 from *P. protegens* Pf-5 integrated into MCS with restriction enzymes SacI and PstI	This work
pSEVA434-*cscY*	Derivative of pSEVA434; gene PFL_3239 from *P. protegens* Pf-5 integrated into MCS with restriction enzymes SacI and XbaI (vector cut with SacI and SpeI)	This work
pTn7-M-*cscRABY*	Derivative of pTn7-M; *cscRABY* gene cluster transferred to the MCS from pSEVA221-*cscRABY* with restriction sites SacI and XbaI	This work
pRK600	Helper plasmid for conjugation; ColE1 *ori*; Cm-resistance; RK2 (mob^{+} tra^{+})	Kessler *et al.*, 1992
pTnS-1	Helper vector for Tn7-transposition containing Tn7-specific transposase	Choi *et al.,* 2005

Table S2 | Bacterial strains that were used in this work

Bacterial strain	Genotype	Function	Source
E. coli DH5***α*** ***λ****pir*	F⁻ ***λ*** ⁻ *endA*1 *glnX44*(AS) *thiE1* *recA1* *relA1* *spoT1* *gyrA96*(Nal^R) *rfbC1* *deoR* *nupG* ***Φ***80(*lacZ****Δ***M15) ***Δ***(*argF-lac*)U169 *hsdR17*(r_K^- m_K^+), ***λ*** *pir* lysogen	Cloning host	Laboratory collection
P. putida KT2440 EM178	Derivative of KT2440; ***Δ***PP3849-PP3920 (prophage 1) ***Δ***PP3026-PP3066 (prophage 2) ***Δ***PP2266-PP2297 (prophage 3) ***Δ***PP1532-PP1586 (prophage 4)	Expression host; production strain	CNB, Victor de Lorenzo, Madrid
E. coli HB101	F⁻ ***λ***⁻ h*sdS20*(r_B^- m_B^-) *recA13* *leuB6*(Am) *araC14* Δ(*gpt-proA*)62 *lacY1* *galK2*(Oc) *xyl-5* *mtl-1* *thiE1* *rpsL20*(Sm^R) *glnX44*(AS)	Helper strain for conjugation	Boyer and Roulland-Dussoix, 1969
Pseudomonas protegens Pf-5	wildtype	Donor for the *cscRABY* gene cluster	Kirsten Jung, LMU, Munich

Table S3 | Oligonucleotides used for PCR reactions in this study with name, sequence and function. Start and stop codons are underlined, restrictions sites are in presented in bold

Name	Sequence	Function
fwP_I_scr_P_pro	TTTT**GAGCTC**ATTGGCTAACACGACTCACG	Amplification of *cscRABY* from *P. protegens* Pf-5
rvP_scr_P_pro	TTTT**GGTACC**TGCTAGGCG**TCTAGA**ACCAG	
fwP_seq_cscRABY_1	TCACGCAATTGCTCAGTACC	Sequencing of pSEVA221 containing *cscRAB*(*Y*) genes
fwP_seq_cscRABY_2	AAGCTATAGGCTCCGTCATC	
fwP_seq_cscRABY_3	TGACCATGATCCAGTTCACG	
fwP_seq_cscRABY_4	CCGAATAGGGATGATGTTGG	
fwP_seq_cscRABY_5	GATGCGCACGAACATGATG	
fwP_seq_cscRABY_6	CCGGCGTAGACATAAATTGC	
fwP_seq_cscRABY_7	AAGTCCAGTTGCTAACCAG	
fwP_seq_cscRABY_8	TCTGCATTGGCTAGACAGAG	
rvPrimer_LUK1	GGATCTATCAACAGGAGTCCAAG	
seq-pSEVA-224-rv	ATCCAGATGGAGTTCTGAGG	
Tn7_fw	AACAGGCTTATGTCAAGACGTC	Outer primer for verification of correct insertion of pTn7-M into *att*Tn7-site of *P. putida*
